# Breast calcification micromorphology classification

**DOI:** 10.1259/bjr.20220485

**Published:** 2022-07-25

**Authors:** Robert Scott, Iain Lyburn, Eleanor Cornford, Pascaline Bouzy, Nicholas Stone, Charlene Greenwood, Ihsanne Bouybayoune, Sarah Pinder, Keith Rogers

**Affiliations:** 1 Cranfield Forensic Institute, Cranfield University, Swindon, United Kingdom; 2 Gloucestershire Hospitals NHS Foundation Trust, Cheltenham, United Kingdom; 3 School of Physics and Astronomy, University of Exeter, Exeter, United Kingdom; 4 School of Chemical and Physical Sciences, Keele University, Staffordshire, United Kingdom; 5 School of Cancer and Pharmaceutical Sciences, King’s College London, London, United Kingdom

## Abstract

**Objectives:**

The importance of consistent terminology in describing the appearance of breast calcifications in mammography is well recognised. Imaging of calcifications using electron microscopy is a globally growing field of research. We therefore suggest that the time is ripe to develop a lexicon of terms for classifying the micromorphology of breast calcifications.

**Methods:**

Calcifications within a wide range of histological sections of breast tissue, both benign and malignant, were imaged by Scanning Electron Microscopy (SEM). These images were examined, and the micromorphology of calcifications present was grouped to create a classification system.

**Results:**

Based on the appearance of the calcifications observed, we propose five main categories for classification of the micromorphology of breast calcifications, namely, Dense Homogenous, Punctulate, Banded, Spongy and Aggregate.

**Conclusions:**

Use of the descriptive categories outlined here will help to ensure consistency and comparability of published observations on the micromorphology of breast calcifications.

**Advances in knowledge:**

This is the first time a lexicon and classification system has been proposed for the micromorphology of breast calcifications, as observed by scanning electron microscopy of histological sections. This will facilitate comparability of observed relationships between micromorphology, mammographic appearance, chemistry and pathology.

## Introduction

As use of mammography expanded rapidly in the 1980s, it quickly became apparent that there was a need for standardisation of terminology in reporting.^1^ Systems such as BI-RADS^1^ would be considerably handicapped if radiologists were to use different terms for the same things, or worse, the same terms for different things. The BI-RADS lexicon of terms, together with pictorial guidelines for their application ensures consistency. The same principles apply to other visual classification systems.

In recent years, there has been a substantial intensification in research on the microscopic physico-chemical characteristics of breast calcifications.^2–7^ In particular, characterisation of pathological calcifications using scanning electron microscopy (SEM) is an increasingly active research field.^8^ Recent publications have applied a range of micromorphology descriptors to breast calcifications imaged using SEM. For instance, similar micron-sized particles have been described as “spherules”,^4^ “punctate particles”,^5^ or “nano and micro spherical particles”.^9^ Other micromorphological descriptors have included “agglomerations”^4^ and “aggregates”.^5^ As in mammography reporting, consistency of descriptors between research groups is also important in establishing relationships between morphology, composition and pathology. In particular, morphology descriptions can help to shed light on mechanisms of formation and growth of pathological calcifications, and may form the basis of diagnostic or prognostic tools.^8^

## Methods

Cases were selected from the Gloucestershire Hospitals NHS Foundation Trust diagnostic archive and from King’s Health Partners Cancer Biobank under NHS HRA/HCRW Ethics Approval (REC reference 20/NW/0057). The former consists of 42 core biopsies from a sequential series identified by the presence of calcifications on a mammogram; the latter consists of a series of 20 surgical excision specimens also selected for the presence of calcifications. In total, there were 28 benign, 9 indeterminate and 25 malignant (*in-situ* or invasive) cases. A series of sections was taken from each of the blocks. One section from each block was mounted on a 12.5-µm polyolefin membrane stretched across an aluminium alloy ring and held in place with a Viton O-ring, as previously described.^10^ These were examined in a Hitachi SU3500 scanning electron microscope in low vacuum mode, using a backscattered electron detector. A chamber pressure of 60 Pa proved sufficient to prevent charging. A significant advantage of mounting sections on a hydrocarbon substrate such as this polyolefin membrane is that calcifications show up very clearly due to atomic number contrast intrinsic to backscattered SEM. This makes it simple to locate all the calcifications in a section. Whole-slice images were stitched together from a matrix of images using Microsoft Image Composite Editor. A total of 88 calcified areas were then imaged at a higher magnification and classified.

## Results and proposed classification

We have grouped micromorphology into the following classes:

### Dense homogenous

**Figure 1.**
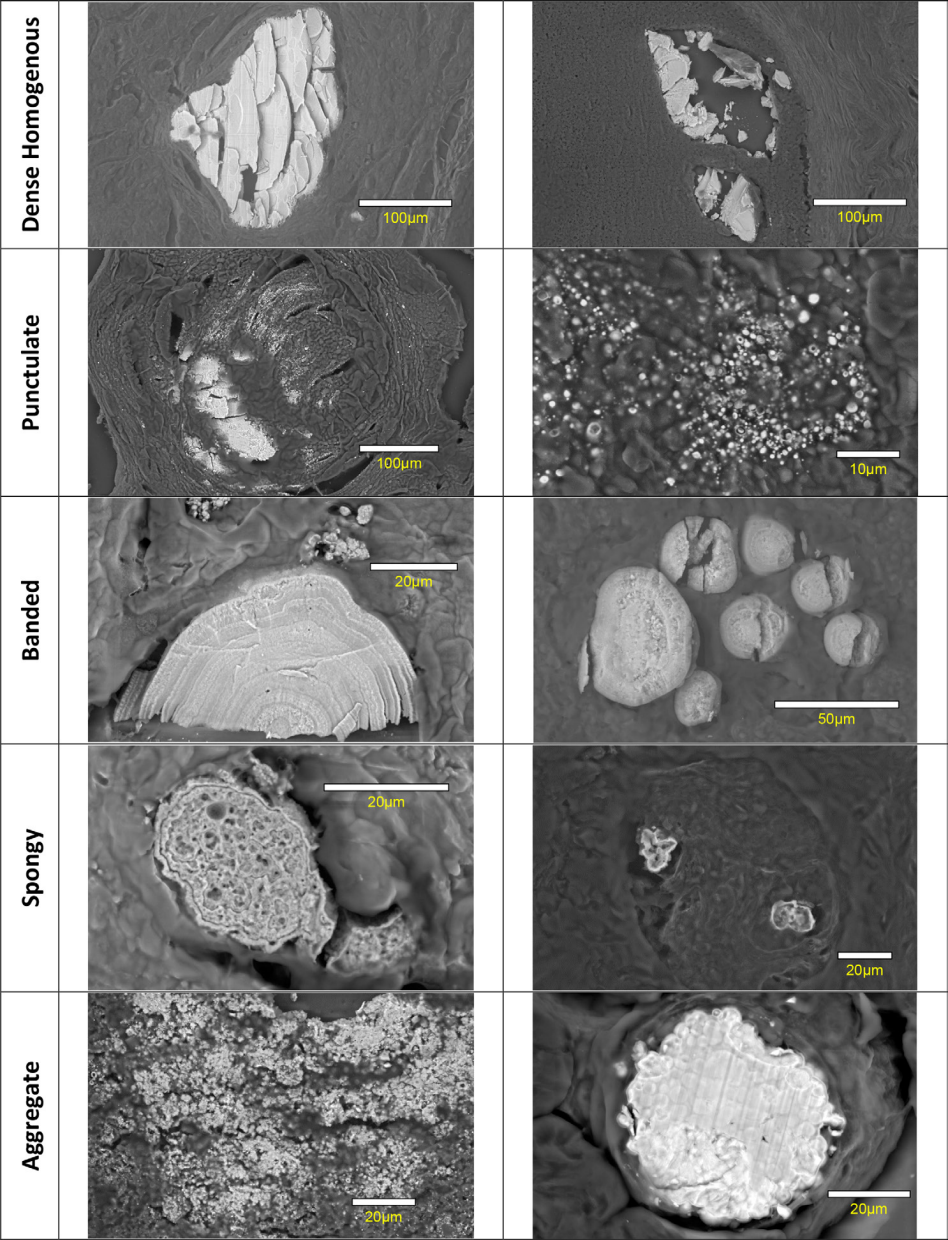
SEM Images showing two examples typical of the five proposed morphology classifications.

This category describes calcifications with dimensions in the range of tens to hundreds of micrometres, without obvious internal structure such as banding. The word “solid” has been avoided since this is a term used to describe the architectural pattern of the proliferation in DCIS.^11^ Note that this type of calcification is frequently extensively fractured by the microtome blade on sectioning. The dense homogenous nature of the calcifications can readily be observed from the nature of the fragments. The two calcifications shown the Dense Homogenous category in Figure 1 consist of calcium phosphate (Type II) and calcium oxalate (Type I), respectively. The latter appear to be more prone to brittle fracture and disintegration on sectioning.

### Punctulate

This category describes separated calcifications which are typically spheroidal with a diameter<10 µm. Punctulate means “minutely punctate”^12^ from the Latin *punctulum*, the diminutive of *punctum* (point). This appropriately distinguishes the term from the BI-RADS classification of “punctate”, since the point-like entities observable in mammography are one or two orders of magnitude larger. These micron-sized spheroidal calcified particles have previously been observed in breast tissue under a variety of names.^4,5,9^ The Punctulate category in Figure 1 shows areas of punctulate calcifications near to a dense homogenous calcification, and a higher magnification image revealing concentric rings in some particles. These rings have been observed in this type of punctulate particle in both breast^4,5^ and vascular^13,14^ calcifications.

### Banded

These are typically a few tens of µm in size and exhibit distinct concentric layers, consistent with growth rings. In some cases, the rings extend to the centre, and in other cases, there appears to be a “geode” core. The latter could be an artefact of a microtome cut that does not pass through the core of the calcification. This type of macroscopic concentric-layered structure is frequently observed in kidney stones and is a characteristic of eight of the subtypes within the seminal classification system developed by Michel Daudon.^15^ Note that these calcifications are substantially different in size and distribution to the punctulate particles, which can also display a concentric ring structure.

### Spongy

A largely continuous matrix containing pores, as distinct from an aggregate of particles. In many cases, these appear to be approximately cell-sized (~20 µm).

### Aggregate

This is a broad category describing calcifications composed of an aggregate of particles, in which the particles have fused partially or completely into a continuous mass. It may be useful to add subcategories to distinguish between the sizes and morphologies of the constituent particles, and the looseness of the aggregation. Aggregates of punctulate particles have previously been observed in breast^4,5^ and vascular^13,14^ calcifications.

### Specimen pathology

All five morphologies have been observed in specimens with an overall pathology opinion of benign, indeterminate and malignant. In particular, it is notable that punctulate particles are not restricted to malignant specimens, as has previously been suggested,^9^ but were found in 7 out of 28 benign specimens, cf. 2 out of 25 malignants.

## Discussion and conclusion

Other morphologies have been observed in isolated cases, but were not deemed to merit a category unless and until they have been observed more frequently. These include calcifications surrounded by one or more poorly calcified layers, or calcifications with a banded internal structure and a loose acicular layer on the outside, reminiscent of a rapidly formed precipitate. The diversity of morphologies observed suggests a variety of formation mechanisms at work. Correlating micromorphology with the wide range of benign and malignant conditions which can form microcalcifications promises to shed light on these mechanisms. This is of particular interest for lesions of uncertain malignant potential. Use of the descriptive categories outlined here should help to ensure comparability of observed relationships between micromorphology, mammographic appearance, chemistry and pathology.
